# In ovo injection of cinnamon or clove alters the physiology and growth of broilers in a hot tropical environment

**DOI:** 10.1093/tas/txad036

**Published:** 2023-03-28

**Authors:** Oluwaseun Ayomide Akosile, Olajide Mark Sogunle, Bukola Majekodunmi, Oyegunle Emmanuel Oke

**Affiliations:** Department of Animal Physiology, Federal University of Agriculture, Abeokuta, Nigeria; Department of Animal Production and Health, Federal University of Agriculture, Abeokuta, Nigeria; Department of Animal Physiology, Federal University of Agriculture, Abeokuta, Nigeria; Department of Animal Physiology, Federal University of Agriculture, Abeokuta, Nigeria

**Keywords:** hatching, in ovo, performance, stress, welfare

## Abstract

A study was conducted to evaluate the influence of in ovo feeding of clove and cinnamon on broilers. The experiment used 700 broiler (Ross 308) hatching eggs that were incubated at the recommended temperature of 37.8 °C. On day 17.5 of incubation, 100 eggs were randomly assigned to each of the following seven treatments: uninjected eggs (OE), eggs injected 0.5 mL distilled water (DH), 2 mg of clove, 4 mg of clove, 2 mg of cinnamon, 4 mg of cinnamon, and 3 mg of ascorbic acid (AC). During the posthatch period, the chicks were raised for 56 days. Data on physiological parameters, growth performance, and intestinal histomorphology were collected. Results revealed that the plasma triiodothyronine (T3) of AC and CV2 chicken was higher than the others. Additionally, the plasma malondialdehyde levels of the chickens of AC, CV2, and CM2 were improved significantly (*P* < 0.05). The initial weights of CV2 birds were comparable with AC CV4, CM4, and CM2 birds but heavier than those of OE and DW. The bodyweight gain in the CV2 group was similar to AC, CV4, and CM2 groups but heavier than OE, DW, and CM4 birds. Feed intake of OE and DW groups was similar to AC, CV2, CV4, and CM2 but higher than CM4. The feed conversion ratio of OE and DW chickens was comparable but higher than the value obtained in chickens of other treatments. The intestinal morphology of the birds did not follow a particular trend. The study concluded that the in ovo injection of 2 mg of clove improved broiler birds’ metabolic and antioxidant status at hatch. The high and low doses of clove and the low dose of cinnamon improved the performance of broiler chickens at the market age in a hot tropical environment.

## INTRODUCTION

Climate change is causing more severe and more frequent effects of heat stress due to seasonal changes, and the intensity of this is likely to increase in the years ahead. Neuroendocrine alterations are activated by the hypothalamus when an animal is exposed to stressful conditions, resulting in poor productivity, morbidity, or death. The impact of heat stress is a serious concern in the tropics. Tropical regions are mainly located around the equator, where the ambient temperatures are rarely below 18 °C for the entire year (Sachs, 2001; Habibu et al., 2016; Meteyake et al., 2020; Oke et al., 2021a, 2022). High ambient temperatures encumber broiler production in tropical environments as most farmers rear their birds in open-sided poultry houses with no control over the harsh environmental variables. However, developing countries in the tropics can benefit from the rapid growth of broilers as a partial solution to widespread protein malnutrition.

The broiler industry is susceptible to heat stress, especially in hot climates. Hartlova et al. (2002) indicated that the absence of sweat glands in broilers increases breathing frequency and core temperature, resulting in an increase in alveolar ventilation and cooling of chickens via evaporation. An environmental temperature above 30 °C adversely affects broiler productivity by reducing feed efficiency, weight gain. and feed intake (Borges et al., 2004). When chickens are thermally challenged, oxidative stress occurs because the tissue’s antioxidant capacity is overwhelmed by free radical generation (Wahab et al., 2010). Due to the peculiarity of broiler production in developing countries in the tropics, sustainable interventions are needed to ameliorate the effect of thermal challenges on birds.

To ameliorate the impacts of thermal stress in birds, dietary modifications with commercial feed additives are considered the most practical and favored methods. Phytogenic feed additives rich in antioxidants have been advocated as possible viable options to alleviate the influence of the heat challenges on chickens (Sharbati et al., 2013; Oke et al., 2017, 2021a, 2021b; Oke, 2018; Kpomasse et al., 2021). Hence, broilers’ growth and immunity could also be enhanced when some medicinal feed additives are included in their diets during periods of heat stress. Beneficial effects of medicinal plants on chickens during stress are known (Hossain et al., 2014; Chegini et al., 2018; Oke et al., 2021b) as they are high in aromatic acids and flavonoid which possess antioxidant antifungal, antibacterial, and anti-inflammatory properties (Al-Sultan et al., 2019; Tokofai et al., 2021).

Apart from its digestion- and appetite-stimulant properties (Tabak et al., 1999), cinnamon (*Cinnamomum cassia*) has been shown to contain many essential oils, including l-borneol, eugenol, caryophyllene oxide, cinnamate, cinnamic acid, and cinnamaldehyde (Tung et al., 2008), possessing antioxidant and antibacterial activities (Singh et al., 2007; Qotbi, 2016). Faixová and Faix (2009) indicated that the antioxidant properties of cinnamon are attributed to its polyphenolic and phenolic compounds. Additionally, there is a growing interest in the use of clove bud (*Syzygium aromaticum*) as an effective antioxidant phytogenic additive (Najafi et al., 2010; Agostini et al., 2012; Gaikwad et al., 2019). The bioactive components of clove are eugenyl acetate, α-humulene, caryophyllene, isoeugenol, and eugenol (Jirovetz et al., 2006). Further, the antioxidant properties of eugenol have been documented (Mil-Homens et al., 2012).

Although there is a plethora of findings on the beneficial dietary impacts of these medicinal plants (Coskun et al., 2017; Mokarrami et al., 2020; Oke et al., 2016, 2020), it is not clear whether the in ovo feeding of different doses of cinnamon and clove extracts will confer thermotolerance and improve the performance of broilers in the hot tropical environments. In light of this, this study aimed to investigate the influence of in ovo feeding of cinnamon and clove on antioxidant status, posthath growth performance, and intestinal morphology of broiler chickens.

## MATERIALS AND METHODS

### Preparation of the Extracts

The clove seeds and cinnamon buds were bought from a local store. The description of N’nanle et al. (2017) was followed to obtain the extracts. Briefly, the samples were thoroughly pulverized into powder, sieved with 0.5-mm mesh, and weighed. A sample of 425 g was soaked in a mixture of 2 L ethanol (100%) and 2 L distilled water for 72 h. The mixture was homogenized and sieved. The evaporation of the solution was done using Heating bath B-491 and Rotavapor R-210 as described by the phytochemical screening manual (Debale, 2002).

### Egg Collection, Incubation, and Candling

A reputable breeder farm provided 1,000 fertile eggs from its Ross 308 flock (38 wk old). The eggs were placed randomly in a single-stage incubator setup tray (SY-7680, Sanyuan Incubation Ltd, China). The incubator was configured to keep the environment at a temperature of 37.8 °C and relative humidity of 60%. A total of 700 fertile eggs were selected from the fertile eggs for the in ovo injection procedure on incubation day 17.5.

### Experimental Design

Seven hundred viable eggs in total were weighed and given individual labels. The 700 hatching eggs were allocated to seven treatments (100 eggs in each treatment having five replicates of 20 eggs each: 0 (uninjected eggs; OE), eggs injected with clove extract at 2 and 4 mg per egg (CV2), cinnamon at 2 and 4 mg peregg (CM2), and 3 mg per egg ascorbic acid (AC) dissolved in 0.5 mL of distilled water (CM2) and sterile water (0.5 mL) (DH). A 1-mL syringe with a sterile needle was used for the in ovo feeding of the extracts.

### Egg Injection Procedure

On embryonic day 17.5, in ovo feeding was carefully done through the air chamber of the incubating hatching eggs. Prior to in ovo injection, the sites of injection on the broad ends of the eggs were cleaned with methylated spirit to avoid contamination. The eggs were drilled above the air space with a sterile needle (18G.) and sealed immediately after the in ovo injection with sterile paraffin wax. The hatching eggs were then transferred to the hatching basket according to the treatment.

### Posthatch Management

The chicks hatched were raised in a disinfected poultry pen until day 56 in line with the in ovo treatments. Upon arrival in the experimental pen, the chicks were tagged, weighed, and replicated, and were reared on an open-sided deep litter housing type having wood shavings as litter materials. There were 70 birds in each treatment, having five replicates of 14 chickens each. The temperature of the poultry pen was maintained between 32 and 33 °C for 7 d. The chickens were fed according to the nutrient requirement of broilers as prescribed by NRC (1994). The average relative humidity and ambient temperature recorded (at 1 pm) during the experiment were 73.4% and 29.5 °C, respectively.

### Data Collection

#### Plasma thyroid hormones, superoxide dismutase, and malondialdehyde.

Samples of blood were taken from four randomly selected chickens per replicate at hatch and 56th day via the brachial vein into ethylene diaminetetraacetic acid (EDTA) anticoagulant and immediately mixed gently to avoid clotting. After centrifuging the heparinized blood for 15 min (3,500 rpm), the plasma was collected and malondialdehyde (MDA) concentrations and superoxide dismutase (SOD) activity was measured as using the methods of Zheng et al. (2020). As described by Tachibana (2007), plasma triiodothyronine concentrations were assayed using immunoenzymatic ELISA kits (CTK Biotech, San Diego, CA). The biochemical indices were determined using the method of Oke et al. (2021b).

#### Determination of physiological indices.

The birds were gently captured randomly from each replicate to determine their rectal temperature. A digital thermometer was gently inserted into the rectum of the birds, following the description of Yahav and McMurtry (2001). Heart rate (HR) was determined using a stethoscope. Body surface temperature (wing and eye) was determined using an infrared thermometer. Data on body surface temperature, HR, and rectal temperature were collected from 10 chicks per treatment (2 chicks per replicate) weekly.

#### Growth performance.

The body weights of the chickens were determined using a sensitive scale (Camry EK 5055-005, Zhongshan Camry Electronic Co. Ltd., China) in each replicate every week. Feed intake and weight gain were recorded on a replicate basis throughout the experiment. The feed conversion ratio was calculated as the ratio of the feed consumed and the weights gained by the chickens.

#### Hematolgogical parameters.

Samples of blood were collected from two birds per replicate on day 56 to determine the hematological parameters. The samples were collected from the brachial vein into heparinized tubes, and the parameters were determined according to the methods of Zanu et al. (2020).

#### Histomorphology of the small intestine.

Fragments of approximately 4 to 5 cm (in length) were taken from the middle region of the jejunum, duodenum, and ileum sections. The excised fragment from each bird was washed in saline (0.9% NaCl) to remove intestinal content and then immersed in natural formaldehyde solution (10% buffered) for fixation. Each segment of the intestine was opened and washed in saline solution, fixed in Bouin solution for 24 h on polystyrene sheets, and was analyzed following the description of Dahlhoff (2008). Morphological examination of the small intestine was done using a light microscope (at 40X magnification) that captured images and a system that analyzed computerized images. The height of eight intact well-oriented villi and the depth of the crypt were determined from each intestinal cross-section as described by Cheled-shoval et al. (2011). Villus height (µm) was measured as the tipoff of the villus crypt junction, and crypt width was taken as the midline of the villus. The mucosa wall thickness was measured from the muscularis layer to the serosa.

### Statistical Analysis

Using the SAS (2008) statistical program, the analysis of variance was performed on the data obtained using a completely random design. Tukey’s HSD was used to separate the means (*P* < 0.05).

## RESULTS

### Physiological Responses at Hatch

The physiological indices of the chicks at hatch are shown in Table 1. There was a similarity in the SOD and thyroxine of the birds at hatch. However, the plasma T3 of the birds of AC and CV2 was similar but higher than those of other treatments whose values were comparable. Also, the plasma MDA of the birds of OE, DH, CV4, and CM4 was higher than those of CV2, CM2, and AC chicks, whose values were not significantly different.

**Table 1.** Physiological responses of chickens to in ovo cinnamon and clove at hatch

Parameter	OE	DH	AC	CV2	CV4	CM2	CM4	SEM	*P* value
SOD	1.63	1.42	2.28	2.25	2.10	1.48	1.75	0.160	0.6747
T_3_	3.03^*b*^	3.01^*b*^	3.92^*a*^	3.68^*a*^	3.21^*b*^	3.16^*b*^	3.09^*b*^	0.068	0.0001
T_4_	9.58	10.57	10.08	11.10	10.80	10.53	12.35	0.356	0.5635
MDA	1.42^*a*^	1.49^*a*^	0.71^*b*^	0.79^*b*^	1.51^*a*^	0.88^*b*^	1.48^*a*^	0.069	0.0001

^*a*,*b*:^Different superscripts signify difference along the row at *P* < 0.05. AC = 3 mg ascorbic acid; CM4 = 4 mg cinnamon; CM2 = 2 mg cinnamon; CV4 = 4 mg clove; CV2 = 2 mg clove; DH = eggs injected with distilled water; OE = uninjected eggs.

### Growth Performance

Responses of broiler chicken to in ovo feeding of aqueous extracts of clove and cinnamon on growth performance are shown in Table 2. The initial weights of CV2 and AC birds were comparable with CV4, CM4, and CM2, but were higher (*P* < 0.05) than OE and DH. The body weight gain of birds in the CV2 group was similar to AC, CV4, and CM2 groups but higher than OE, DH, and CM4 chickens. The feed intake of the OE and DH group was similar to AC, CV2, CV4, and CM2, but significantly (*P* < 0.05) higher than CM4. The feed conversion ratio of OE and DH birds was (*P* < 0.05) higher than the values obtained in the birds of other treatment groups.

**Table 2.** Responses of broilers to in ovo cinnamon and clove on performance

Parameter	OE	DH	AC	CV2	CV4	CM2	CM4	SEM	*P* value
I WG, g	33.75^c^	36.75^bc^	45.50^a^	43.50^a^	38.75^abc^	42.25^ab^	42.25^ab^	1.78	0.0001
F W G, g	2236.00^b^	2255.00^b^	2449.25^ab^	2567.50^a^	2374.50^ab^	2335.50^ab^	2203.25^b^	93.06	0.0002
B W G, g	2202.25^b^	2218.25^b^	2404.75^ab^	2524.00^a^	2335.75^ab^	2293.25^ab^	2161.00^b^	93.47	0.0002
F I, g	5638.40^a^	5583.30^a^	5355.40^ab^	5513.70^ab^	5255.40^ab^	5276.10^ab^	5128.50^b^	188.80	0.0078
F C R, g/g	2.56^a^	2.51^a^	2.22^b^	2.18^b^	2.25^b^	2.30^b^	2.30^b^	2.08	0.0001

^a,b,c^:Different superscripts signify difference along the row at *P* > 0.05. AC = 3 mg ascorbic acid; BWG = body weight gain; CM4 = 4 mg cinnamon; CM2 = 2 mg cinnamon; CV4 = 4 mg clove; CV2 = 2 mg clove; DH = eggs injected with distilled water; FCR = feed conversion ratio; FI = feed intake; FWG = final weight gain; IWG = initial weight gain; OE = uninjected eggs.

### Physiological Parameters at the Starter and Finisher Phases

In ovo feeding of the aqueous extract did not have a significant (*P* > 0.05) influence on wing temperature, eye temperature, and HR at the starter phase (Table 3). However, the rectal temperature of CM4 birds was similar to the other treatment group, except for CM2, which had a lower value. At the finisher phase, rectal temperature, wing temperature, and HR were not influenced by in ovo injections. However, the eye temperature of OE was similar to other treatment groups but higher than CM4.

**Table 3.** Responses of broiler chicken under hot-humid season to in ovo cinnamon and clove on physiological parameters at starter and finisher phases

Starter phase									
Parameters	OE	DH	AC	CV2	CV4	CM2	CM4	SEM	P value
Rectal temperature (°C)	41.15^ab^	41.27^ab^	41.07^ab^	41.22^ab^	41.12^ab^	41.00^b^	41.67^a^	0.28	0.055
Wing temperature (°C)	38.00	37.50	38.75	38.00	37.75	37.00	38.50	0.91	0.177
Eye temperatures (°C)	36.00	36.00	35.50	36.25	34.00	36.75	35.75	1.33	0.172
Heart rate (beats/min)	336.00	339.00	324.00	322.50	319.50	337.50	339.00	14.31	0.226
Finisher phase									
Rectal temperature (°C)	41.45	41.45	41.72	41.12	41.32	41.47	41.77	0.357	0.207
Wing temperature (°C)	36.55	36.25	36.78	37.08	36.20	36.65	36.80	0.782	0.363
Eye temperatures (°C)	37.45^a^	35.80^ab^	36.67^ab^	36.45^ab^	36.20^ab^	36.57^ab^	35.60^b^	0.751	0.044
Heart rate (beats/min)	356.12	370.17	356.77	358.45	368.50	360.13	361.80	12.05	0.564

^a,b,c^Different superscripts signify difference along the row differ at *P* < 0.05. AC = 3 mg ascorbic acid; CM4 = 4 mg cinnamon; CM2 = 2 mg cinnamon; CV4 = 4 mg clove; CV2 = 2 mg clove; DH = eggs injected with distilled water; OE = uninjected eggs.

### Hematological Parameters

The chickens’ RBC, PCV, hemoglobin, eosinophil, lymphocyte, monocyte, neutrophil, and platelet were not influenced (*P* > 0.05; Table 4). However, the CV2 birds had a comparable WBC value with AC, CV4, OE, and CM2 but higher than DH and CM4. The heterophil of the OE birds was similar to CV2, DH, CV4, and CM4 but higher (*P* < 0.05) than AC and CM2. The H:L ratio of OE birds was similar to DH, CV4, CM2, and CM4 but higher (*P* < 0.05) than AC and CV2 birds.

**Table 4:** Responses of broiler chickens under hot-humid season to in ovo cinnamon and clove on hematological parameters

Parameters	OE	DH	AC	CV2	CV4	CM2	CM4	SEM	P value
PCV (%)	27.67	25.00	26.67	26.00	23.66	30.67	26.33	3.17	0.264
RBC(×10^6^/µL)	1.29	1.21	0.98	1.53	1.36	1.48	1.21	0.34	0.531
WBC(×10^6^/µL)	2.61^ab^	1.16^c^	2.51^abc^	3.10^a^	2.66^ab^	2.08^abc^	1.316^b^	0.50	0.002
HM(g/dl)	12.17	11.10	11.90	11.67	10.63	13.23	11.80	1.10	0.202
Eosinophil (%)	0.33	1.66	0.33	0.66	1.33	1.33	1.33	0.78	0.269
Lymphocyte (%)	66.67	63.67	70.33	73.33	68.00	67.00	70.67	4.90	0.609
Monocyte (%)	1.67	1.66	2.333	2.33	1.33	2.67	1.66	0.95	0.592
Neutrophil (%)	31.33	32.33	34.33	29.00	29.33	29.00	27.33	4.01	0.423
Heterophil (%)	31.67^a^	30.00^ab^	27.00^b^	28.33^ab^	28.00^ab^	27.00^b^	29.33^ab^	1.38	0.015
Platelet (%)	64.00	78.00	60.33	66.00	106.00	106.00	88.00	19.66	0.046
H/L (%)	0.47^a^	0.47^ab^	0.38^b^	0.38^b^	0.41^ab^	0.41^ab^	0.41^ab^	0.03	0.013

^*a*,*b*^:Different superscripts signify difference along the row at *P* < 0.05. AC = 3 mg ascorbic acid; CM4 = 4 mg cinnamon; CM2 = 2 mg cinnamon; CV4 = 4 mg clove; CV2 = 2 mg clove; DH = eggs injected with distilled water; OE = uninjected eggs.

### Serum Biochemical Parameters

Responses of broiler chickens to serum biochemical parameters at the finisher phase are shown in Table 5. In ovo feeding of the extracts did not significantly affect total protein, creatinine, ALT, albumin, globulin, and triglyceride at the finisher phase. However, the serum glucose (*P* < 0.05) of the chickens on OE and DH was similar to those of CV4, CV2, and CM4, but higher than the birds in AC and CV2.

**Table 5.** Responses of broiler chicken to in ovo cinnamon and clove on serum biochemical parameters

Parameters	Total protein mg/dL	Creatinine mg/dL	ALT (U/L) mg/dL	Albumin mg/dL	Globulin mg/dL	Glucose mg/dL	Triglyceride mg/dL
OE	6.26	0.3	55.5	3.76	2.4	143.67^a^	88.86
DH	7.3	0.35	45	3.73	3.4	142.67^a^	96.93
AC	7.2	0.45	60.5	3.53	3.7	98.50^b^	91.36
CV2	6.76	0.45	48	3.63	3.2	95.65^b^	93.83
CV4	6.3	0.3	56.5	3.3	3.06	125.67^ab^	101.4
CM2	5.93	0.35	65.5	3.56	3.06	134.00^ab^	84.06
CM4	6.8	0.45	51	3.56	3.03	123.33^ab^	101.1
SEM	0.61	0.01	1.75	0.23	0.53	14.9	6.93
*P* value	0.129	0.087	0.452	0.33	0.087	0.027	0.069

AC = 3 mg ascorbic acid; CM4 = 4 mg cinnamon; CM2 = 2 mg cinnamon; CV4 = 4 mg clove; CV2 = 2 mg clove; DH = eggs injected with distilled water; OE = uninjected eggs.

### Intestinal Histomorphology

Table 6 shows the responses of broilers to in ovo feeding of cinnamon and clove extracts on ileal, jejunal, and duodenal histomorphology at day 56. Duodenal, jejunal, and ileal villus heights were similar (*P* < 0.05) across the treatments. Ileal villus width not influenced by the treatments. The duodenal villus width of CV2 and AC chickens was higher (*P* < 0.05) than those of OE, DH, CV4, CM2, and CM4. Jejunal villus width of AC birds was comparable with those of OE, DH, CV2, CV4, and CM2 but significantly higher (*P* < 0.05) than CM4. Duodenal, jejunal, and ileal crypt depth showed comparable values across all treatments, except for CV2 and AC birds, which had significantly lower (*P* < 0.05) values. For jejunal crypt depth, CM4 and OE birds had comparable values with CV4, DH, and CM2, but were significantly higher (*P* < 0.05) than AC and CV2. The ileal crypt depth of DH and CM4 was similar to OE and CV4 but significantly higher (*P* < 0.05) than AC, CV2, and CM4 birds.

**Table 6.** Responses of broilers to *in ovo* cinnamon and clove on intestinal histomorphology on day 56

Parameters	OE	DH	AC	CV2	CV4	CM2	CM4	SEM	*P* value
Duodenum									
VH (μm)	1,288.67	1,336.33	1,330.67	1,433.33	1,158.00	1,262.00	1,228.00	103.86	0.111
VW (μm)	117.66^b^	123.33^b^	141.67^ab^	156.66^a^	118.33^b^	118.00^b^	123.00^b^	11.84	0.007
CD (μm)	156.00^abc^	181.00^a^	133.33^c^	145.00^bc^	170.00^ab^	154.67^abc^	180.33^a^	11.60	0.001
Jejunum									
VH (μm)	1,623.00	1,440.30	1,743.70	1,814.70	1,592.70	1,417.00	1,481.00	175.25	0.097
VW(μm)	184.67^ab^	195.67^ab^	229.33^a^	199.00^ab^	184.67^ab^	188.33^ab^	188.33^b^	27.18	0.117
CD (μm)	262.67^a^	173.67^ab^	162.67^b^	163.67^b^	176.33^ab^	177.67^ab^	258.33^a^	2.36	0.007
Ileum									
VH (μm)	2,140.00	2,059.6	2,115.4	2,365.5	1,907.5	1,621.7	1,954.5	269.39	0.104
VW (μm)	242.74	277.82	278.27	213.34	222.03	213.34	205.52	42.89	0.581
CD (μm)	229.70^ab^	365.61^a^	211.46^b^	213.43^b^	236.28^ab^	200.86^b^	313.50^a^	50.37	0.009

^a,b.c^Means having different superscript in the row differ significantly at *P *< 0.05. AC = 3 mg ascorbic acid; CD = crypt depth; CM4 = 4 mg cinnamon; CM2 = 2 mg cinnamon; CV4 = 4 mg clove; CV2 = 2 mg clove; DH = eggs injected with distilled water; OE = uninjected eggs; VH = villus height; VW = villus width.

## DISCUSSION

This study evaluated the in ovo feeding with clove and cinnamon on broilers’ perinatal and posthatch growth in a hot climate. During incubation, various physiological processes of chick embryos are regulated by thyroid hormones during the transition to the lung from allantoic respiration and hatching events (McNabb, 2000; Reyns et al., 2003; de Smit et al., 2008). To meet the need for more energy during hatching, T3 plays a crucial role (Piestun et al., 2009). The higher plasma T3 recorded in the hatched chicks of CV2 eggs in the present study can be ascribed to the clove’s bioactive constituents for inducing the chicks’ energy metabolism (El-Kholy et al., 2019). Similar to the CV2 chicks, the improved plasma T3 observed in the AC birds is congruent to the findings of El-Kholy et al. (2019), who demonstrated that the hatched chicks from vitamin C in ovo injection had higher plasma T3 than the control group. The significant increase in plasma T3 may be attributed to the enhanced activity of the thyrotrophic axis of the birds. The similarity of the plasma SOD of the birds recorded in this study aligns with the observations of Zhu et al. (2019), who demonstrated that in ovo ascorbic acid, injection did not affect the plasma SOD of chickens.

A nutritional supplement that could potentially attenuate oxidative stress could ameliorate the adverse effects of stress on the posthatch growth performance of broilers (Ebeid et al., 2013; Hajati et al., 2014), and the protection provided by antioxidants at hatching is essential for the survival of chickens in the early period following hatching (Surai et al., 2016). The lower MDA of CV2 and AC chickens in this study indicates a better antioxidant status. The plasma triiodothyronine of the birds may explain the improved antioxidant status as Laurberg et al. (2005) and Lin et al. (2008) demonstrated that antioxidant enzymes are largely controlled by thyroid hormones. The similarity in the plasma MDA of the birds treated with cinnamon extract and the control group in the present study suggests that the bioactive compounds of the extract did not upregulate the antioxidant activity.

There is a nexus between the performance of broilers and intestinal microbiota, which may be modified to provide favorable conditions for increased growth. A healthy gastrointestinal tract is critical for nutrient digestion and absorption (Mitchell et al., 2006; Hajati et al., 2014). Eugenol (in clove) has antioxidant properties comparable to synthetic antioxidants (Dorman et al., 2000). The amelioration of oxidative stress at the late stage of incubation might have led to the higher weight gain and performance of the chicks (Choe et al., 2019). The improved performance of the birds in CV2 and AC in this study could be attributed to improved intestinal development. Antioxidants offer oxidative defense mechanisms to the gut of developing embryos, shielding them from free radicals (Surai, 1999; Choe et al., 2019). The improved final body weight of CV2 and AC birds in this study indicates that the clove’s bioactive constituents enhanced broiler performance. Dietary administration of clove has been reported to improve the body weight of birds (Ertas et al., 2005; Najafi and Torki, 2010). However, Al-mufarrej et al. (2019) reported that the inclusion of dietary supplementation of clove at 2% and 6% resulted in a decline in body weight. Our findings in this study revealed that there was a higher feed intake by CV2 and AC birds. There is an inconsistency in the dietary effect of clove on the feed intake of chickens. Milind and Deepa (2011) reported a higher feed intake, while other authors reported that there was no effect (Mukhtar, 2011; Petrovic et al., 2012; Mahrous et al., 2017), whereas other authors reported a decline in the feed intake (Mohammadi et al., 2014; Al-mufarrej et al., 2019). The discrepancy in the results may be attributed to variations in management, breeds of chickens, and methods of processing phytogenic feed additives. Nevertheless, the in ovo injection of CV2 and AC resulted in a higher feed intake. There is a need to take caution in comparing in ovo with dietary supplementation, as in ovo injection involves the assimilation of substances at different growth phases. The improved performance of the CV2 birds in this trial is consistent with the results obtained in dietary treatment (Park, 2008; Gbenga et al., 2009). Most of the hematological parameters of the chickens in this study were somewhat similar, indicating the safety of the phytogenic additives via in ovo route. This corroborates the recent findings of Oke et al. (2021b), which showed that in ovo black cumin did not alter the hematology of broilers.

The present study indicates that the glucose levels were substantially different in CL2 and vitamin-C-treated birds, with a lower value than the other groups. High blood glucose has been established during fasting and stressful conditions (Maduka et al., 2015). The lower glucose levels suggest that the birds offered clove 2 mg and AC injections had better thermotolerance under the hot season in combating the effect of heat stress. According to Zhang et al. (2015), phytochemicals can prevent hypoglycemia by modulating glycosidase and lipase activities, lowering glycemic levels, enhancing pancreatic function, and having a synergistic effect.

The small intestine is a highly specialized organ for nutrition breakdown and absorption. It is a barrier between the host’s exterior and internal environments (Sobolewska et al., 2017). Digestive enzyme secretion increases as crypts go deeper (Xu et al., 2003). As a result, the villi surface area, crypt depth, and the ratio of villi height to crypt depth are typical indicators of intestinal functional and developmental conditions (Xu et al., 2003). Increasing any of these morphometric characteristics will improve the brush border membrane’s digestive and absorptive capacities. The observed intestinal morphology of the broilers in the present study suggests that the modulation of the gut did not follow a particular trend, and the similarity in some of the intestinal morphological parameters of the birds may be due to the effects of the in ovo feeding fading with time. The depth of the crypts is one of the factors determining the health and function of the intestine in chickens, and their size can indicate the intensity of intestinal epithelial cell regeneration (Samanya and Yamauchi, 2002). The duodenal and ileal crypt depth of CV2 and AC birds in this study suggests a healthy gut with an efficient tissue turnover, indicating better nutritional absorption. This observation corresponds to the better growth performance of the birds.

In conclusion, our findings in the present study have shown that in ovo injection of cinnamon and clove aqueous extracts at low levels (2 mg) improved the plasma triiodothyronine, antioxidant status and the final body weights than the control group. Although the duodenal villus width of the CV2 chickens was better than the control birds, the different doses of the extract did not elicit a specific trend in the intestinal morphology of the broilers. The chickens offered in ovo feeding of 2 mg cinnamon competed favorably with AC in plasma MDA, the rectal temperature at the starter phase, and FCR. It is suggested that in ovo injection of cinnamon or clove at 2 mg per egg could be beneficial for the performance of broilers in tropical environments.
